# The Relationship between Cadherin Polymorphisms and the Risk of Delayed Encephalopathy after Acute Carbon Monoxide Poisoning in the Chinese Han Population

**DOI:** 10.1155/2022/3155703

**Published:** 2022-01-30

**Authors:** Xuejiao Liu, Jiao Zeng, Xiaoli Zhang, Jiapeng Gu, Fan Zhang, Yongkai Han, Ping Zhang, Wenqiang Li, Renjun Gu

**Affiliations:** ^1^The Second Affiliated Hospital of Xinxiang Medical University, Henan Mental Hospital, 453002, China; ^2^Henan Key Lab of Biological Psychiatry of Xinxiang Medical University, International Joint Research Laboratory for Psychiatry and Neuroscience of Henan, 453002, China

## Abstract

**Objective:**

The purpose of this study was to analyze the relationship between cadherin gene single-nucleotide polymorphisms (SNPs) and the risk of delayed encephalopathy after acute carbon monoxide poisoning (DEACMP).

**Materials and Methods:**

A total of 416 patients with DEACMP and 754 patients with acute carbon monoxide poisoning (ACMP) were recruited. We used the Sequenom MassARRAY® system to detect cadherin gene SNPs related to DEACMP. Using different genetic analysis models, we evaluated the relationship between the cadherin gene polymorphisms and risk of DEACMP.

**Results:**

We found that rs1944294 in the N-cadherin (CDH2) gene showed significant differences in genotype frequencies between the two groups under codominant and dominant inheritance models. Similarly, rs2513796 in the cadherin-17 (CDH17) gene showed significant differences under the codominant, dominant, and overdominant genetic models. And the T allele frequency of rs1944294 in the DEACMP group was significantly higher than that in the ACMP group (*P* = 0.023).

**Conclusions:**

Cadherin gene SNPs (rs1944294, rs2513796) are associated with an increased risk of DEACMP in the Chinese population.

## 1. Introduction

China remains a largely agricultural country with 450 million people living in rural areas relying on charcoal fires for heating in winter. This can result in acute carbon monoxide poisoning (ACMP) which is also the major cause of emergency patient visits to primary hospitals in winter [1]. ACMP is a common occupational and living environment-related risk with delayed encephalopathy after ACMP (DEACMP) representing one of its most common and serious complications. The incidence of DEACMP in affected patients ranges from 13% to 50%, and its mortality rate can be as high as 31% [2]. After ACMP and a period of false recovery (2-60 days), DEACMP manifests in patients as a series of neurological and psychiatric disorders including affective disorders, personality changes, cognitive dysfunction, gait disorders, incontinence, and Parkinson's syndrome. Examination by computed tomography (CT) or magnetic resonance imaging (MRI) can show bilateral symmetric foci of the globus pallidus and/or extensive demyelination changes in the white matter of the brain [3].

The pathogenesis of DEACMP is complex and uncertain with present understanding of the disease involving changes at the genetic, molecular, and cellular levels which accompany the anatomical changes. Various insults including ischemia and hypoxia, oxidative stress, inflammation, and neurotransmitter toxicity result in changes in the nutritional microenvironment of brain cells, resulting in cell death by apoptosis and necrosis, causing damage to important functional areas of the brain, and ultimately manifesting as DEACMP [4–7]. Interestingly, the incidence of DEACMP varies greatly among patients of similar age and gender, suggesting that environmental as well as genetic factors play roles in disease etiology. Indeed, our group previously explored the contribution of genetics to the pathogenesis of DEACMP. We conducted genome-wide association studies (GWAS) of single-nucleotide polymorphisms (SNP) on peripheral blood samples of 175 DEACMP and 244 ACMP Chinese patients. Our results identified genes such as neurexin 3, Parkinson disease 2, myelin basic protein (MBP), neuron-specific enolase (NSE), and leucine-rich repeats and calponin homology domain containing 1 (LRCH1) which were associated with DEACMP [8–12]. In this study, we now extend this analysis to consider the contribution of genetic variation in cadherin family genes to the development of DEACMP.

Cadherins are a family of cell adhesion molecules that play a variety of roles in the development and maintenance of the vertebrate central nervous system. These roles include regulation of the division of neural tube regions, neuronal migration, gray matter differentiation, neural circuit formation, spinal cord morphology and synapse formation, and remodeling along with the participation in intracellular signaling pathways related to neuropsychiatric diseases [13]. Among the different cadherin genes, neural or N-cadherin also known as cadherin-2 (CDH2) has been shown to mediate axon sorting [14] and to promote the recruitment and migration of neural precursor cells from neural stem cell nests in the subventricular zone into demyelinating lesions [15]. In addition, it has been reported that N-cadherin is overexpressed in experimental models of autoimmune encephalomyelitis (EAE) where it affects the migration of oligodendrocytes [16]. Also, in the adult central nervous system, N-cadherin expression can be recapitulated postnatally under pathological conditions, and thus, the N-cadherin may initiate remyelination in autoimmune-mediated inflammatory demyelination [17]. It is presumed that cadherin or its signaling pathway can be closely related to the pathogenesis of DEACMP.

A better understanding of the susceptibility of an individual to DEACMP may conceivably provide guidance for clinical treatments. Currently, there are no reports concerning the relationship between genetic variations in cadherin genes and the pathogenesis of DEACMP. With this in mind, we conducted a case-control study in the Chinese population to evaluate the correlation between single-nucleotide polymorphisms in cadherin genes and the development of DEACMP.

## 2. Materials and Methods

### 2.1. Study Participants

A total of 1203 patients, including 416 DEACMP patients and 754 ACMP patients, were recruited from the emergency and neurology departments of 11 hospitals in Henan, including the Second Affiliated Hospital of Xinxiang Medical University, from November 2006 to April 2019. The selection of the ACMP patients was performed according to the Chinese occupational ACMP diagnostic criteria (GBZ23-2002). For the DEACMP patients, the diagnostic criteria included the occurrence of one of the following clinical manifestations after 2-60 days of false recovery of consciousness from acute carbon monoxide poisoning: (1) mental and consciousness disorders in a state of dementia, delirium, or decortex; (2) Parkinson's syndrome manifestations in extrapyramidal neurological disorders; (3) pyramidal nerve damage, such as hemiplegia, positive pathological reflex, or urinary incontinence; and (4) focal dysfunction of the cerebral cortex, including aphasia, blindness, and/or secondary epilepsy. Examinations performed included the following: (1) CT or MRI of the head to find bilateral symmetric foci of the globus pallidus and/or extensive demyelination changes in the white matter of the brain and (2) electroencephalogram (EEG) examination to find moderate and severe abnormalities.

The two groups of patients were matched in the age, gender, and educational level. In order to achieve comparable age matching between the patient groups, only patients over 40 years old were selected given that DEACMP patients under 30 years are rare. Furthermore, since the false recovery period of DEACMP can be as long as 60 days, a follow-up for more than 90 days was applied to all ACMP patients. Peripheral blood samples were collected in anticoagulation vacuum tubes at 6:00~8:00 am after overnight fasting for the DEACMP patients and within 24 hours after being fully awake for the ACMP patients. All the blood samples were labeled and stored in a −80°C refrigerator. The research plan was approved by the ethics committee of the Second Affiliated Hospital of Xinxiang Medical University and all participating hospitals and research institutions. All the participants signed written informed consent to participate in the study.

### 2.2. Genotyping

Genomic DNA was extracted from the peripheral blood using the TIANGEN kit (DP304, Beijing Tiangen Biotechnology Co., Ltd.). The Sequenom MassARRAY® platform was used to perform SNP genotyping of all the samples. First, the amplification primers required for the 4 SNP sites of cadherin according to the dbSNP database were designed and sequenced, and the site-specific PCR was obtained by multiplex PCR amplification. Then, ABI Veriti-384 PCR was used for the initial multiplex PCR amplification and extension. After resin purification, MassARRAY Nanodispenser (Agena, Inc.) was used to transfer the PCR products to a 384-well biochip. Finally, the genotype and alleles were detected by using the MassARRAY Analyzer 4.0 and analyzed by using MassARRAY TYPER4.0. As a result, 4 SNPs were genotyped in all samples.

### 2.3. Statistical Analysis

The data were analyzed using the SPSS 25.0 statistical software. The independent sample *t*-test was used for age comparisons while the chi-square test was used to compare sex and education levels. The chi-square goodness-of-fit test was used to analyze whether the distribution of genotypes conformed to the Hardy-Weinberg equilibrium law, and binary logistic regression analysis was used to analyze the allele and genotype frequencies between the groups and calculate 95% confidence intervals. A *P* < 0.05 was considered to represent statistically significant differences.

## 3. Results

### 3.1. Baseline Characteristics

We analyzed the genotypes of 416 DEACMP patients (over 40 years old) and 754 ACMP patients. After sequencing, four synonymous coding SNPs (rs1899646, rs1944294, rs2513796, and rs6028103) were detected in cadherin genes.  Table 1 shows the physical location and gene frequency of the four cadherin gene SNPs. The distribution of the SNPs was 413 and 751 patients for rs1899646, 413 and 754 patients for rs1944294, 412 and 750 patients for rs2513796, and 414 and 754 patients for rs6028103 in the DEACMP and ACMP groups, respectively. There was no difference in age, gender, and educational level of DEACMP and ACMP patients among the four SNP loci (*P* > 0.05; Table 2). In addition, there was no deviation in the genotype distribution of the DEACMP and ACMP patients from the tested polymorphism relative to the Hardy-Weinberg balance (Table 3).

### 3.2. Association Analysis between rs1899646 (CDH2), rs1944294 (CDH2), rs2513796 (CDH17), and rs6028103 (CDH4) Polymorphisms and DEACMP

The genotype and allele frequencies of the four SNPs assessed in this study are shown in Table 4. The results of the binary logistic regression analysis, performed to compare the genotype distribution of the DEACMP and ACMP groups, indicated that rs1944294 showed a significant difference in genotype frequency between the DEACMP and ACMP groups in codominant (AA vs. AT) and dominant (AA vs. AT+TT) models (*P* = 0.048; *P* = 0.024). In addition, the rs1944294 allele frequency difference was statistically significant between the two patient groups (*P* = 0.023). The T allele may be a potential risk allele of DEACMP.

In contrast, rs1944294 was not associated with an increase in the risk of DEACMP in the recessive (AA+AT vs. TT) and overdominant (AA+TT vs. AT) models (*P* > 0.05). Similarly, rs2513796 was associated with DEACMP sensitivity in codominant (GG vs. GA), dominant (GG vs. GA+AA), and overdominant (GG+AA vs. GA) models (*P* = 0.013, *P* = 0.022, and *P* = 0.013, respectively). In the recessive model (GG+GA vs. AA), there was no statistical association between rs2513796 and DEACMP risk (*P* > 0.05). The other two sites, rs1899646 and rs6028103, were not associated with DEACMP risk in all four models (*P* > 0.05).

## 4. Discussion

Our study foremost reveals for the first time that the rs1944294 (CDH2) polymorphism in codominant and dominant models and rs2513796 (CDH17) in codominant, dominant, and overdominant models were associated with an increased risk of DEACMP. Moreover, the T allele of the rs1944294 (CDH2) polymorphism may be a risk factor for DEACMP. Thus, gene polymorphisms in different cadherin genes may underlie the molecular basis of DEACMP development. Notably, recent research has shown that N-cadherin (CDH2) promotes the recruitment and migration of neural progenitor cells from neural stem cell niches in the subventricular zone (SVZ) to demyelinating lesions [15]. In addition, it has been reported that N-cadherin is the cause of poor astrocyte migration promotion properties, and the reduction of intercellular adhesion may affect the proliferation of oligodendrocyte progenitor cells in demyelinating multiple sclerosis lesions [16]. Hochmeister et al. also suggested that the expression of N-cadherin can be induced under pathological conditions in the adult central nervous system, where it plays an important role in the initiation of autoimmune inflammatory demyelination [17]. Since N-cadherin is broadly implicated in homeostasis in different brain pathologies, it could be reasonably envisaged that the functional status of N-cadherin or other cadherins would play an important role in the pathological changes associated with DEACMP, for example, the extensive demyelination of white matter.

Mechanistically, the function of different cadherin-related transmembrane glycoproteins has been closely linked with the activation of Rho-GTPases through the regulation of guanine nucleotide exchange factors (GEFs) [18, 19]; these in turn affect the downstream ROCK signaling pathway. The G protein-coupled receptor facilitates the conversion of Rho proteins into their active Rho-GTP (mainly RhoA), facilitating binding to ROCK kinase though the Rho binding domain to expose its catalytic activity center and thereby activate ROCK kinase. Consequently, this results in ROCK dephosphorylating and inactivating myosin light chain phosphatase (MLCP) which then attenuates the phosphorylation of the myosin light chain (MELC) [20]. In the context of nerve regeneration, axon growth is inhibited, thereby blocking the repair of demyelination damage [21]. Rho proteins are involved in a variety of physiological processes, such as actin cytoskeleton reorganization, cell movement and adhesion, cell morphology changes, cell proliferation, cell growth and apoptosis, and gene transcription [22]. These proteins can also participate in inflammation by regulating the activation of p38 MAPK [23]. For example, MAPK activation can regulate the expression of proinflammatory cytokines, including IL-1, IL-6, and TNF-*α*, as well as cyclooxygenase-2 (COX2) and inducible nitric oxide synthase (iNOS) [24]. The onset of DEACMP is closely related to many immune cells and inflammatory factors, such that the infiltration of T cells, B cells, macrophages, and neutrophils and the cascade-like release of inflammatory factors such as interferon-*γ*, interferon-*α*, IL-6, IL-8, and others participate in the occurrence of DEACMP [8]. Thus, it is plausible that alterations in cadherin function through genetic variation results in changes in ROCK and MAPK signaling that impact the repair of demyelination damage and inflammatory responses during DEACMP pathogenesis.

Finally, we must consider the limitations of this study. Firstly, our selection of four SNP sites in cadherin genes represents only a small component of the DEACMP candidate-associated genes. Furthermore, the sample size is presently limited and insufficient to clarify causal mechanisms in a disease such as DEACMP with highly complicated pathogenesis. Furthermore, DEACMP is considered to result from the combined effects of genetic and environmental risks, and the latter has not factored into our risk model. Nevertheless, future high-throughput analysis technologies are expected to identify the most meaningful gene loci and provide more complete evidence of genetic mechanisms involved in the pathogenesis of DEACMP.

## Figures and Tables

**Table 1:** Physical locations of the 4 cadherin gene SNPs.

Variant	Chrom	Position	MAF
rs1899646 (CDH2) C/G	18	27933109	0.155 (C)
rs1944294 (CDH2) A/T	18	28036487	0.206 (T)
rs2513796 (CDH17) G/A	8	94130191	0.215 (A)
rs6028103 (CDH4) C/T	20	61311096	0.304 (C)

**Table 2:** Demographic variables of DEACMP and ACMP patients genotyped for the 4 SNPs (rs1899646, rs1944294, rs2513796, and rs6028103 polymorphisms).

Characteristic	rs1899646 (CDH2) DEACMP	ACMP	Statistics	*P* value	rs1944294 (CDH2) DEACMP	ACMP	Statistics	*P* value	rs2513796 (CDH17) DEACMP	ACMP	Statistics	*P* value	rs6028103 (CDH4) DEACMP	ACMP	Statistics	*P* value
	*N* = 413	*N* = 751			*N* = 413	*N* = 754			*N* = 412	*N* = 750			*N* = 414	*N* = 754		
Age (y) ± SD (range)	64.80 ± 11.67	65.13 ± 15.95	*t* = 0.361	0.718	64.89 ± 11.67	65.13 ± 15.93	*t* = 0.261	0.794	64.82 ± 11.66	65.09 ± 15.93	*t* = 0.298	0.765	64.92 ± 11.69	65.11 ± 15.92	*t* = 0.214	0.831
Total	413	751			413	754			412	750			414	754		
Male	242	434	*χ* ^2^ = 0.071	0.790	242	435	*χ* ^2^ = 0.089	0.765	242	432	*χ* ^2^ = 0.141	0.707	242	435	*χ* ^2^ = 0.064	0.801
Female	171	317			171	319			170	318			172	319		
Education level																
Uneducation	130	200	*χ* ^2^ = 3.3	0.188	130	200	*χ* ^2^ = 3.4	0.181	130	198	*χ* ^2^ = 3.7	0.155	130	200	*χ* ^2^ = 3.4	0.176
Primary school	141	264	48		140	265	23		140	264	28		142	265	80	
Middle school	142	287			143	289			142	288			142	289		

**Table 3:** Results of the Hardy-Weinberg equilibrium test for genotype distributions of cadherin polymorphisms.

SNP	Genotypes	Risk allele	Risk minor allele frequency DEACMP/ACMP	Actual value	Test value
rs1899646 (CDH2)	CC	C		21/30	*χ* ^2^ = 0.006/0.003 *P* = 0.936/0.956
	CG		0.224/0.199	143/239	
	GG			249/482	

rs1944294 (CDH2)	AA	T		217/344	*χ* ^2^ = 0.349/2.020 *P* = 0.555/0.155
	AT		0.271/0.316	168/343	
	TT			28/67	

rs2513796 (CDH17)	GG	A		214/337	*χ* ^2^ = 1.398/2.704 *P* = 0.237/0.100
	GA		0.288/0.320	159/346	
	AA			39/67	

rs6028103 (CDH4)	CC			57/99	
	CT	C	0.384/0.375	204/368	*χ* ^2^ = 0.713/1.257 *P* = 0.398/0.262
	TT			153/287	

**Table 4:** Association analysis of cadherin polymorphisms under different genetic models and DEACMP risk.

SNPs	Genetic models		DEACMP	ACMP	*P*_*obs*_	OR (95% CI)
rs1899646 (CDH2)	Allele	(G/C)	641/185	1203/299	0.157	1.161 (0.944-1.428)
	Codominant	(GG/CG/CC)	249/143/21	482/239/30	0.264/0.303	1.158 (0.895-1.498)/1.355 (0.760-2.416)
	Dominant	(GG/CG+CC)	249/164	482/269	0.189	1.180 (0.922-1.511)
	Recessive	(GG+CG/CC)	392/21	721/30	0.386	1.287 (0.727-2.279)
	Overdominant	(GG+CC/CG)	270/143	512/239	0.330	1.135 (0.880-1.463)

rs1944294 (CDH2)	Allele	(A/T)	602/224	1031/477	0.023^∗^	0.804 (0.666-0.971)
	Codominant	(AA/AT/TT)	217/168/28	344/343/67	0.048^∗^/0.088	0.776 (0.604-0.998)/0.662 (0.413-1.063)
	Dominant	(AA/AT+TT)	217/196	344/410	0.024^∗^	0.758 (0.596-0.964)
	Recessive	(AA+AT/TT)	385/28	687/67	0.210	0.746 (0.472-1.179)
	Overdominant	(AA+TT/AT)	245/168	411/343	0.113	0.822 (0.644-1.048)

rs2513796 (CDH17)	Allele	(G/A)	587/237	1020/480	0.106	0.858 (0.712-1.033)
	Codominant	(GG/GA/AA)	214/159/39	337/346/67	0.013^∗^/0.692	0.724 (0.561-0.933)/0.917 (0.596-1.410)
	Dominant	(GG/GA+AA)	214/198	337/413	0.022^∗^	0.755 (0.593-0.961)
	Recessive	(GG+GA/AA)	373/39	683/67	0.763	1.066 (0.704-1.613)
	Overdominant	(GG+AA/GA)	253/159	404/346	0.013^∗^	0.734 (0.574-0.937)

rs6028103 (CDH4)	Allele	(T/C)	510/318	942/566	0.677	1.038 (0.872-1.236)
	Codominant	(TT/CT/CC)	153/204/57	287/368/99	0.769/0.692	1.040 (0.802-1.349)/1.080 (0.738-1.580)
	Dominant	(TT/CT+CC)	153/261	287/467	0.709	1.048 (0.818-1.343)
	Recessive	(TT+CT/CC)	357/57	655/99	0.759	1.056 (0.744-1.500)
	Overdominant	(TT+CC/CT)	210/204	386/368	0.878	1.019 (0.802-1.295)

## Data Availability

The experimental data used to support the findings of this study are included within the article.
